# Lateral Habenula Responses During Eye Contact in a Reward Conditioning Task

**DOI:** 10.3389/fnbeh.2022.815461

**Published:** 2022-03-14

**Authors:** Hyunchan Lee, Okihide Hikosaka

**Affiliations:** Laboratory of Sensorimotor Research, National Eye Institute, National Institutes of Health (NIH), Bethesda, MD, United States

**Keywords:** lateral habenula, eye contact, reward, primate, electrophysiology

## Abstract

For many animals, social interaction may have intrinsic reward value over and above its utility as a means to the desired end. Eye contact is the starting point of interactions in many social animals, including primates, and abnormal patterns of eye contact are present in many mental disorders. Whereas abundant previous studies have shown that negative emotions such as fear strongly affect eye contact behavior, modulation of eye contact by reward has received scant attention. Here we recorded eye movement patterns and neural activity in lateral habenula while monkeys viewed faces in the context of Pavlovian and instrumental conditioning tasks. Faces associated with larger rewards spontaneously elicited longer periods of eye contact from the monkeys, even though this behavior was not required or advantaged in the task. Concurrently, lateral habenula neurons were suppressed by faces signaling high value and excited by faces signaling low value. These results suggest that the reward signaling of lateral habenula may contribute to social behavior and disorders, presumably through its connections with the basal ganglia.

## Introduction

Eye contact is a key element of social interactions between conspecifics and even across species. This is especially true in primates (Mosher et al., 2011). Abnormal patterns of eye contact are a common behavioral symptom in autism spectrum disorder (Szatmari et al., 2016). Social behavior is an intrinsic source of natural reward and can release reward-associated neuromodulators (e.g., dopamine and serotonin) (Krach et al., 2010; Trezza et al., 2010; Dölen et al., 2013). However, there have been few studies on how eye contact behavior relates to reward and reinforcement learning.

In the primates, interactions are driven by long-term relationships and are thus necessarily shaped by past experience with outcomes of prior interactions. Although some studies have suggested that aversive feelings such as fear and avoidance lead to gaze aversion (Schneier et al., 2011), this effect is not found in all cases (Wieser et al., 2009). Given the complexity of affiliative and antagonistic behaviors in primate societies, we hypothesized that neuronal networks comprising the reward system might regulate eye contact together.

What are brain structures likely to contribute to social aspects of gaze behavior? Previous work in the lab established the role of several basal ganglia structures for learning the emotional value of non-social objects (Hikosaka et al., 2006, 2019). Among them, the lateral habenula (LHb) is highly sensitive to the emotional significance and interacts with brainstem areas and basal ganglia which can control the release of reward-associated neuromodulators (e.g., dopamine and serotonin) (Matsumoto and Hikosaka, 2007; Hikosaka, 2010; Hu et al., 2020) that could be important in establishing relationships and engaging in social behavior. We thus hypothesized that LHb plays a role in establishing eye contact based on prior emotional experience.

## Results

**FIGURE 1:**
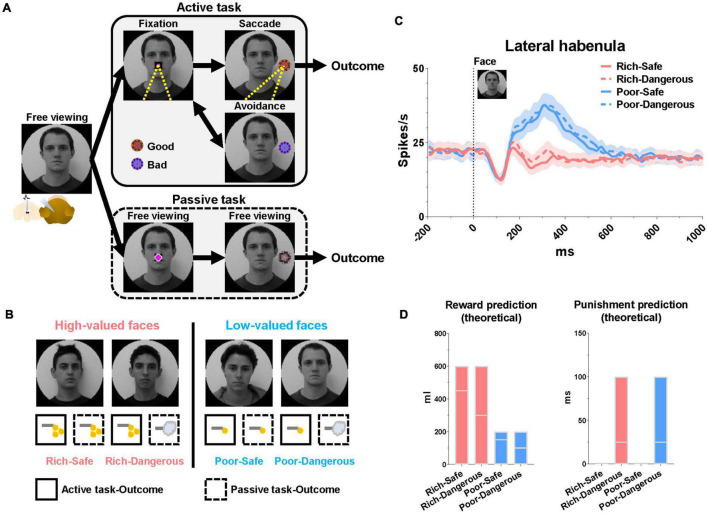
Lateral habenula responses to high- and low-valued faces. **(A)** Face-based procedure with two distinct behavioral tasks (active and passive). **(B)** Examples of face images signaling different conditions. Rich and Poor faces were associated with high-valued and low-valued rewards, respectively. Dangerous faces (but not Safe faces) were associated with punishment in the passive mode. **(C)** Population LHb responses to the faces during the free viewing period before the start of each task. Data were from both monkeys and all recorded LHb neurons averaged across entire sessions. **(D)** Theoretical reward and punishment predictions at face onset. Floating bars indicate the minimum to the maximum value of outcomes in each trial at the face onset. The lines in the bars indicate the mean value of the reward (Rich-Safe, 450 μl; Rich-Dangerous, 300 μl; Poor-Safe, 150 μl; Poor-Dangerous, 100 μl) and punishment (Rich-Safe, 0 ms; Rich-Dangerous, 25 ms; Poor-Safe, 0 ms; Poor-Dangerous, 25 ms) in each trial at the face onset. The mean values were determined by the average of all combinations of outcomes in the active and passive conditions (Supplementary Tables 1,2).

To test this hypothesis, we recorded 33 LHb neurons in two monkeys (15 in monkey CH and 18 in monkey KI) performing an active/passive task in which different face images signaled four different emotional contexts (Figures 1A,B). The entire population of recorded cells in the LHb showed consistent responses to the value of each reward stimuli in the task procedure. We thus expect the significant population of cells in the LHb would play a role in modulating the gaze duration/eye contact. In this task, emotional context varied from trial to trial, governed by the possibility of large or small juice rewards (“Rich” or “Poor” contexts) and also by the occurrence or absence of an aversive airpuff stimulus (“Dangerous” or “Safe” contexts). The airpuff was only delivered in the passive task.

The face images were assigned four distinct emotional contexts based on task mode (active or passive) and amount × probability of the outcome. All face stimuli maintained consistent emotional contexts in both active and passive tasks (Figure 1B). (1) Rich-Safe (Rwd +, Pun−): monkeys experienced big rewards in both tasks. (2) Rich-Dangerous (Rwd +, Pun +): monkeys experienced big rewards in the active task but punishment in the passive task. (3) Poor-Safe (Rwd−, Pun−): monkeys experienced small rewards in both tasks. (4) Poor-Dangerous (Rwd−, Pun +): monkeys experienced small rewards in the active task and punishment in the passive task. At the time of face stimulus onset, the expected reward amount was higher in the Rich-context (Rich-Safe and Rich-Dangerous) compared with Poor-contexts (Poor-Safe and Poor-Dangerous) (Figure 1D and Supplementary Table 1).

Each trial started with the appearance of a face image, the identity of which informed the monkey whether the context of the current trial was Rich-Safe, Rich-Dangerous, Poor-Safe, or Poor-Dangerous (Figure 1A). After a free viewing of the face for 1 s, an active or passive cue appeared at the center of the screen and respective tasks diverged. In the active task, the monkeys were required to fixate their gaze (700 ms) on the active cue. After the fixation, one of the “good” or “bad” objects appeared. The monkeys were then required to make a saccade to “good” fractal objects to obtain a juice reward and avoid gazing at “bad” objects to preserve the possibility of reward later in the trial. After a “bad” object, there was necessarily a “good” object. When the “bad” objects appeared, after the avoidance for 1 s, the active cue re-appeared and “good” objects appeared. The monkeys then could make a saccade to the “good” object to obtain a juice reward. The good and bad objects were different between 4 faces or scenes. Each face or scene environment contained five different fractals (5 fractals/environment × 16 environments/set = total 80 fractals/set) for “good” and “bad” objects in the active task and “100,” “50,” and “0%” objects in the passive task. Monkeys could learn these objects within five blocks (Active task, 192 trials/block + Passive task, 192 trials/block = total 384 trials/block). After five blocks of the learning, the gaze pattern and neuronal responses of monkeys were constantly discriminative to the reward value of stimuli (Rich vs. Poor environments, Good vs. Bad, 100 vs. 0% objects in Rich contexts). The passive task was a Pavlovian conditioning procedure entailing three conditioned stimuli (CS) comprising different outcome probabilities (100, 50, or 0%). In the passive task, the monkeys were not required to make a saccade to any object and could freely observe this. Outcomes in the passive task occurred irrespective of the monkey’s behavior; thus, the resulting pattern of eye movements can be considered a form of natural viewing.

### Reward History Modulates LHb Activity and Eye Contact During Face Viewing

**FIGURE 2:**
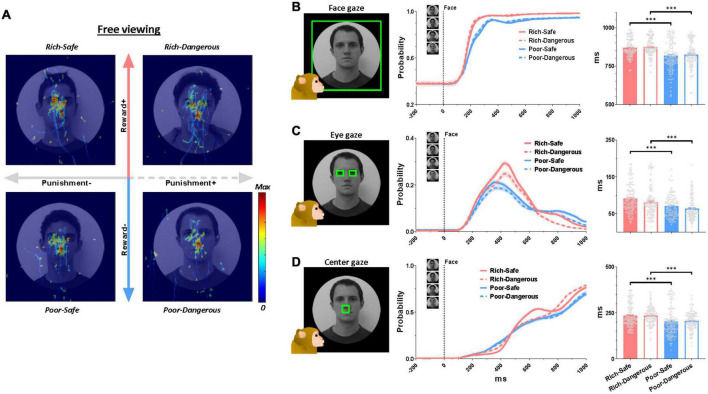
Eye gaze to high- and low-valued faces. **(A)** Normalized eye scan patterns during the free viewing period. Hot colors indicate higher gaze probability. **(B–D)** Left, green boxes indicate regions of interest. Middle, probability of gaze within the said region over all trials ever recorded in the active and passive conditions during the entirety of 138 behavioral sessions. Right, quantification of gaze on the face regions. The average duration of gaze inside the region of interest inside the following time windows: gaze on the face, 0-1,000 ms (top row), gaze at eyes, 0-1,000 ms (middle row), gaze at screen center, 600-1,000 ms (bottom row). Data were from both monkeys and all behaviors averaged across entire sessions.

At the start of the trial, LHb neurons were significantly inhibited by Rich faces and excited by Poor faces (Figure 1C). Furthermore, in Rich trials, the monkeys’ gaze consistently dwelled longer on the face images than on Poor trials (irrespective of Danger vs. Safe) (Figure 2A). LHb neurons showed an initial inhibitory response to every face lasting from 50 to 150 ms. On Rich trials, monkeys showed an increase to look at the face at all from 100 to 150 ms after the trial started (Figure 2B). After 150 ms both LHb activity and gaze behavior discriminated between Rich and Poor faces. Specifically, on Rich trials as compared to Poor, the monkeys’ gaze dwelled longer inside rectangular regions around the eye region, evidently for the sake of discerning what conditions were on the menu for the current trial. Eye contact behavior did not differ between Dangerous vs. Safe trials (Figure 2C). Around 450 ms, the monkeys’ gaze shifted from the eyes to the center of the screen where the active or passive cue would appear (Figure 2D). The probability of gaze within the central window was greater on Rich trials than on Poor trials in a window of 600-1,000 ms. This indicates that the monkey was more motivated by Rich context faces to see the upcoming cue and learn whether an active or a passive trial would follow.

### Reward History Modulates LHb Activity and Saccades to Fractal Objects

**FIGURE 3:**
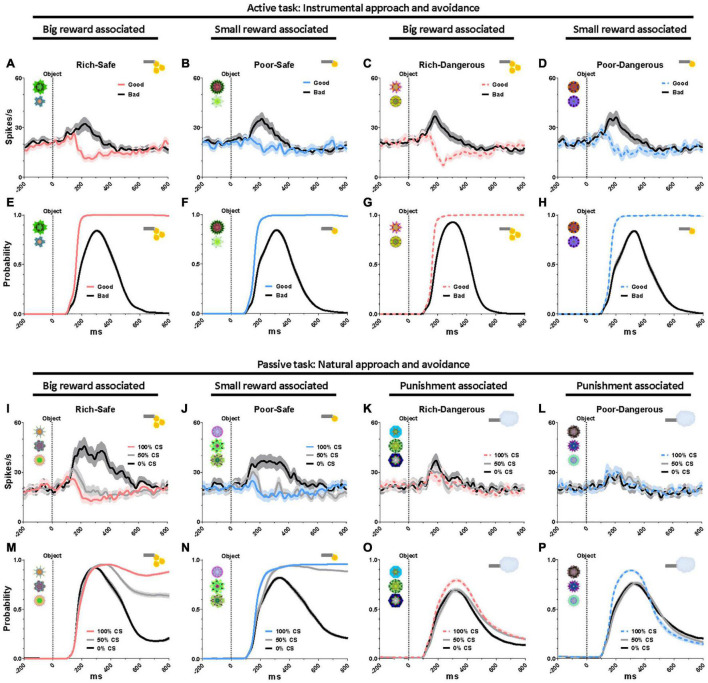
Eye gaze to high- and low-valued objects. **(A–D)** LHb responses to good and bad objects during the active task. **(E–H)** Probability of gaze on good and bad objects during the active task. **(I–L)** LHb responses to the conditioned stimuli (CS) objects during the passive task. **(M–P)** Probability of gaze on the CS objects during the passive task. Data were from both monkeys and all recorded LHb neurons and behaviors averaged across entire sessions.

To assess the impact of task context on reward modulation of LHb neurons over and above the impact of social stimuli, we compared neural responses to saccade targets in the active task and CS fractals in the passive task. In the active task, LHb was suppressed by good objects that monkeys were required to fixate on (> 500 ms), and excited by bad objects that monkeys avoided (Figures 3A–H). In the passive task, Safe contexts, LHb was excited by the 0% CS (signaling a disappointing reward omission) and were inhibited to graded degrees by the 50 and 100% CS (Figures 3I,J). The probability of saccades to CS increased as a function of reward probability (Figures 3M,N). In the Dangerous contexts, LHb was similarly excited (Figures 3K,L) and gaze to CS suppressed by all CS objects regardless of punishment probabilities (Figures 3O,P). Although this finding suggests that reward expectation exerts a more decisive influence on behavior and LHb neurons than punishment expectation, these identical responses of LHb activities and gaze to punishment objects could also have a “warning” role whatever the probability of airpuff occurrence.

## Discussion

### Reward History and Eye Contact: A Perspective of Social Skill/Habitual Behavior

The present study found that the activities of LHb neurons are modulated by the reward history of environmental stimuli (Figure 1C). Moreover, the environments that were associated with greater rewards elicited prolonged gaze periods (Figure 2). This relationship between reward experience and gaze duration was consistently observed in both social (faces, Figure 2) and non-social stimuli (landscape scenes, Supplementary Figures 1A–C). In the social environment, monkeys’ eye contact and face-to-face gaze were strongly modulated by the history of prior reward experiences. Additionally, the pattern of LHb activity and gaze behavior was consistently changed by reward experiences of objects in both natural free viewing (Figures 3M–P, no action required) and instrumental viewing (3E–H, action required).

In real life, many animals have inter-species social gaze/interaction as well as the behaviors within conspecifics. Significantly, the interactions with human are solid and crucial to captive or domestic animals (Tuber et al., 1996; Brosnan and De Waal, 2003; Range et al., 2009; Dettmer et al., 2016). Here, a social bond and emotional history between the animals and humans could play critical roles in the social behaviors of animals beyond their species-specific responses (Stoeger et al., 2012; Katayama et al., 2019). We thus tested LHb activities and monkey’s social gaze using human faces. We then propose that the face and eyes of animals could function as a goal-oriented rewarding object in social contexts and that LHb neurons play an important role in learning this behavior through habitual practice and cultural acclimation.

Consistent with this notion, one study reported that face patches are not observed in newborn primates (Livingstone et al., 2017), and experience with faces is necessary to develop normal face viewing behavior and dedicated face processing modules in the brain (Arcaro et al., 2017). Moreover, in humans, the interpretation of eye contact varies widely across cultures (Uono and Hietanen, 2015). This raises the possibility that the culturally expected pattern of eye contact behavior is a social skill acquired over the course of normal social development. A previous study has reported that macaque monkeys have a strong hierarchical social structure that dominants monopolize 87% of food in the social tolerance test (Burkart and van Schaik, 2013). This finding implies that appropriate social recognition and behaviors based on their social relationship and culture could be critical sources to their living on the social structures.

How then does the brain modulate eye gaze based on emotional histories? We recently suggested that parallel circuits in basal ganglia play an important role in automatic skills and habitual eye movements (Hikosaka et al., 2019). These studies showed that primates spontaneously make saccades to objects associated with reward over the long term. To facilitate neuronal plasticity of basal ganglia neurons and establish the automatic behavior, LHb may play an important role in this form of learning by relaying reward prediction errors signals to dopamine neurons. In turn, these dopamine neurons facilitate synaptic plasticity in basal ganglia neurons, thus paving the way for automatic behavior.

### Hypothetical Network Implementing Lateral Habenula Modulation of Gaze Holding

How might neural activity in LHb lead to sustained eye contact on rewarding objects? A crucial mechanism for stopping eye movements in the brain is modulated by omnidirectional pause neurons (OPN) in the raphe interpositus nucleus (Optican and Pretegiani, 2017). The OPN tonically fire during fixation and abruptly cease their firings before and during a saccade. Then OPN can directly control saccadic eye movements by inhibiting and disinhibiting excitatory/inhibitory burst neurons (EBN/IBN) (Hikosaka and Kawakami, 1977; Igusa et al., 1980; Nakao et al., 1980; Yoshida et al., 1982), premotor neurons of the oculomotor nerve (abducens nerve) (Hikosaka et al., 1977).

**FIGURE 4:**
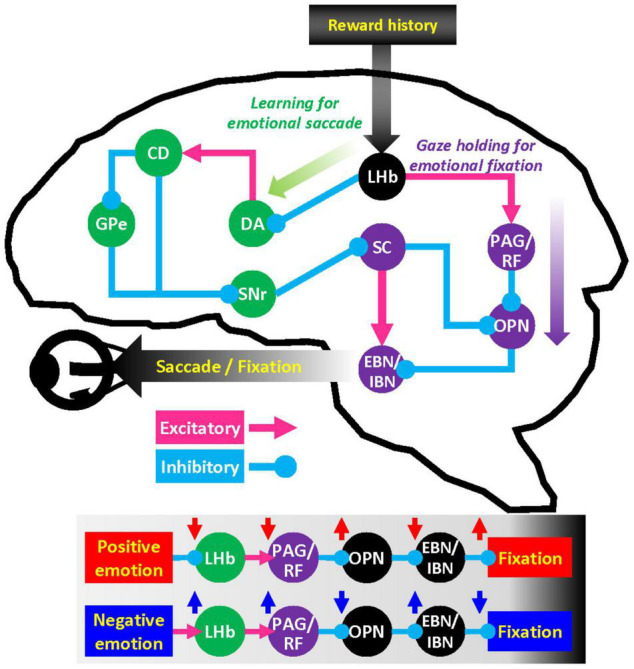
Hypothetical network for the influence of emotion on gaze. LHb neurons regulate dopamine (DA) neurons in substantia nigra pars compacta and ventral tegmental area through rostromedial tegmental nucleus. Dopamine neurons in turn send signals to caudate head (CD) nucleus that projects to superior colliculus (SC) via the external segment of the globus pallidus (GPe) and substantia nigra pars reticulata (SNr). This basal ganglia–SC circuit can modulate learning of goal-directed saccadic eye movements. LHb also has substantial projections to the brainstem structures such as periaqueductal gray (PAG) and reticular formation (RF). These brainstem areas can regulate premotor burst neuron (EBN/IBN) and omnipause neuron (OPN) that control the oculomotor nerve.

In a previous retrograde tracing study in primates, cells projecting to OPN were found in brainstem areas including reticular formation, periaqueductal gray, superior colliculus (SC), and the habenulopeduncular tract (Langer and Kaneko, 1990). SC is a well-studied brain area that projects to OPN (Yoshida et al., 2001; Takahashi et al., 2005) and the premotor burst neurons that control eye movements (Sugiuchi et al., 2005; Izawa et al., 2007; Takahashi et al., 2014). Moreover, the basal ganglia-SC pathway is critical to modulating automatic skills and habitual eye movements established by reward history (Kim et al., 2015; Amita and Hikosaka, 2019; Kunimatsu et al., 2021). LHb neurons access the basal ganglia-SC loop at the level of striatum and dopamine neurons and are thus well-positioned to modulate emotional factors driving learning in oculomotor behavior (Figure 4) (Matsumoto and Hikosaka, 2007; Hong and Hikosaka, 2013). Whereas the outputs of LHb have been extensively studied in midbrain dopamine neurons, LHb projections to other brain stem nuclei are still not clearly appreciated.

Nonetheless, LHb has massive projections to the periaqueductal gray (Li et al., 1993), reticular formation, and parabrachial nucleus (Herkenham and Nauta, 1979). These brainstem areas play an essential role in the motivation of behavior (Kim et al., 2020) as well as direct motor control of facial movement and vocalization (Magoun et al., 1937) that execute quintessentially social gestures. We suggest that this LHb-brain stem pathway may play a pivotal role in gaze holding through the OPN mechanisms and facilitate the execution of social behaviors in general.

### Why Is Eye Contact Important?

Eye contact is a crucial affiliative social behavior that has been reported from the start of infancy (Ferrari et al., 2009). Primates, in particular, possess facial features (e.g., prominent irises and eyebrows, articulate mouth, and facial muscles) optimized to attract gaze and signal intention and feelings (Zhang et al., 2021). More broadly, many animals depend on collective behaviors for survival that must be coordinated and executed at the level of a pack, flock, swarm, or school (Norris and Schilt, 1988; Stander, 1992). Eye contact might support the organization of gregarious behavior in many species.

In addition to eyes and faces, hands are a frequent target of socially guided eye movements (Ninomiya et al., 2020). A previous study showed that, in monkeys raised without exposure to faces, eye movements dwelled longer on human hands than on faces (Arcaro et al., 2017). For captive primates, the human hand might be an object of critical interest for predicting rewards, for instance during enrichment activities or at feeding time.

Like this, there are many social and non-social objects associated with each reward history in real life. Thus, sequential behaviors to multiple objects are often required for attaining goals. For instance, making eye contact can lead to the discernment of social context, which in turn can inform subsequent behavior as warranted by the social goals. In the experiments described here, the monkeys’ eye movements dwelled on the central position as well as the eye regions (Figure 2). This central gaze fixation was longer for Rich faces than for Poor faces (Figure 2D). In our task procedure, the center of the face was the region where the active or passive cue would appear after the free viewing stage (Figure 1A). Thus, it would be one of the significant anticipatory gazes for the upcoming targets (i.e., active or passive cue). Indeed, the monkeys’ gaze acquired the central cue faster in the Rich contexts than in Poor contexts, implying higher motivation on the former trials (Supplementary Figures 1D–H).

In summary, the present study found that LHb neuronal activities represent the reward value of each social and non-social object sequentially and phasically. We thus suggest that the sequential LHb neuronal activities and the gazes of monkeys around the eye region and other prominent reward features in this study might similarly contribute to monkeys’ interactions with other animals like a monkey and human interaction as a sequential goal-directed behavior.

## Materials and Methods

Two adult rhesus monkeys (both male, 8-years-old, 10-12 kg) were used for this study. All animal care and experiment procedures were approved by Animal Care and Use Committee of the National Eye Institute and complied with the Public Health Service Policy on the Humane Care and Use of Laboratory Animals. We recorded 54 single neurons in LHb region around + 7 mm anterior to the interaural plane and + 1 mm from midline using a plastic recording chamber and grid with 1 mm spacing. The recording sites were identified by MRI (4.7 T, Bruker). We then found 33 neurons in the LHb that were sensitive to reward prediction error. The neuronal activities were inhibited by unexpected reward outcomes and excited by reward omission and punishment outcomes as shown in the previous study (Matsumoto and Hikosaka, 2009). The LHb neurons were recorded from only one grid hole at each hemisphere. The LHb neurons were distinguished with the surrounding mediodorsal thalamus region (1 mm away from the LHb neurons) which showed unclear reward prediction error response.

The neurons in both monkeys were recorded with glass-coated electrodes (diameter 0.38 mm, 1 MΩ, Alpha-Omega). The chamber was tilted posteriorly by 8°. The electrode was advanced by an oil-driven micro-manipulator (MO-97A, Narishige). A microelectrode AC amplifier (model 1800; A-M Systems) was used to amplify the neuronal signals (10 k gain) and band-pass filtered from 0.1 to 10 kHz (model 3384; Krohn-Hite). Single neurons were isolated using an online custom voltage- and time-based window discriminator in the software Blip (www.robilis.com/blip/) and collected at 1 kHz along with monkey’s eye position (EyeLink 1000 Plus, SR Research).

The monkeys were trained to perform the face-based active/passive task (five blocks) (Figure 1). They were head-fixed during the task. At the start of a trial, a face (40° × 40°)^1^ represented one of four contexts appeared (Rich-Safe, big reward and no punishment; Rich-Danger, big reward and punishment; Poor-Safe, small reward and no punishment; Poor-Danger, small reward and punishment) (Figure 1B). We used these human face images to investigate the effect of reward history on the social gaze of captive monkeys who are familiar with interaction with human caregivers. On half of the trials (192 out of 384 total trials), landscape scenes were used instead of faces^2, 3^ as non-social stimuli (Supplementary Figure 1). The total of 384 trials was conducted per cell. There was no difference between the tasks when the face stimuli were used and the tasks when scene stimuli were used. After 1 s free viewing of the face/scene stimulus, either an active (Magenta square) or a passive cue (Magenta circle) (2 × 2°) appeared at the center of the screen (Figure 1A middle). The face/scene stimuli stayed on the screen throughout the entire trial (Figure 1A). The shape of the cue indicated whether the trial would proceed as an active or passive task. In the active task, monkeys were required to fixate (700 ms) on the active cue. After the fixation on the active cue, either a good or a bad object appeared on the left or right side of the screen (15 degree). The monkeys could collect the reward by fixating on the good object that appeared for 500 ms. They were required to avoid any bad objects that appeared by not gazing at the object for more than 500 ms. The volume of the juice reward was adjusted depending on whether the trial was Rich (600 μl) or Poor (200 μl). In the active task, reward volume was the same in both the Safe and the Dangerous contexts (Figure 1B). The passive task was a Pavlovian conditioning procedure and required no particular behavior from the monkeys. The monkeys were not needed to fixate on any cue or object in this condition. Each trial of the passive task started with a 1 s free viewing epoch of the face/scene stimulus and was indistinguishable from the preamble to the active task. After the passive cue appeared at the center screen for 1 s, one of three objects (fractal images) appeared in the peripheral region of the face/scene background image. The identity of the fractal stimulus indicated the probability of the reward/punishment outcome (100, 50, and 0%). After 1.5 s, monkeys received a reward (either big or small) in the Rich-Safe and Poor-Safe contexts and received an airpuff (100 ms) in both Rich-Dangerous and Poor-Dangerous according to the probabilities previously foreshadowed by the fractal object. The objects were created using fractal geometry (Yamamoto et al., 2012). Data were analyzed using MATLAB (MathWorks) and Prism8 (GraphPad Software) and presented as mean ± standard error of the mean (SEM). Firing rates were presented by smoothening with a Gaussian kernel (σ = 10 ms). The statistical significances were tested using a one-way analysis of variance (ANOVA) with Tukey *post hoc* test. The statistical tests were performed on groups of cells and behaviors from both monkeys.

## Data Availability Statement

The original contributions presented in the study are included in the article/Supplementary Material, further inquiries can be directed to the corresponding author/s.

## Ethics Statement

The animal study was reviewed and approved by National Eye Institute Animal Care and Use Committee. Written informed consent was obtained from the individual(s) for the publication of any identifiable images or data included in this article.

## Author Contributions

HL and OH conceived, designed, performed the experiments, analyzed the data, and wrote the manuscript. Both authors contributed to the article and approved the submitted version.

## Conflict of Interest

The authors declare that the research was conducted in the absence of any commercial or financial relationships that could be construed as a potential conflict of interest.

## Publisher’s Note

All claims expressed in this article are solely those of the authors and do not necessarily represent those of their affiliated organizations, or those of the publisher, the editors and the reviewers. Any product that may be evaluated in this article, or claim that may be made by its manufacturer, is not guaranteed or endorsed by the publisher.

## Footnotes

1
https://fei.edu.br/c̃et/facedatabase.html

2
https://www.google.com/earth

3
https://openaerialmap.org

## Supplementary Material

Supplementary Figure 1 Responses to high- and low-valued scenes. **(A)** Normalized eye scan patterns during the scene-based task procedure. Hot colors indicate a higher gaze probability. **(B)** Lateral habenula responses to the scenes at the scene onset. **(C)** Probability of gaze on the scene regions. **(D)** Start time of anticipatory gaze on the center region before task cue onset. **(E)** Start time of fixation on the active task cue after the cue onset. **(F)** The rates of trials that monkeys refused to fixate their gaze on the active cue. **(G)** Start time of fixation on the passive task cue after the cue onset. **(H)** The rates of trials that monkeys refused to fixate their gaze on the passive cue.
