# Supplementary material for: Lateral Habenula Responses During Eye Contact in a Reward Conditioning Task
Source: Front Behav Neurosci. 2022 Mar 14;16:815461. doi: 10.3389/fnbeh.2022.815461 (PMC8964066; doi:10.3389/fnbeh.2022.815461)
Supplement: Supplementary file 1 [file Table_1.docx]

**Supplementary Table S1. Theoretical reward prediction**

| **Face/Scene** | **Action Cue** | **Object** | **Outcome** |
| --- | --- | --- | --- |
| Rich-Safe (450 µl) | Active (600 µl) | Bad-Good (600 µl) | 600 µl |
|  |  | Good (600 µl) | 600 µl |
|  | Passive (300 µl) | pRwd 100% (600 µl) | 600 µl |
|  |  | pRwd 50% (300 µl) | 600 µl or 0 µl |
|  |  | pRwd 0% (0 µl) | 0 µl |
| Rich-Dangerous (300 µl) | Active (600 µl) | Bad-Good (600 µl) | 600 µl |
|  |  | Good (600 µl) | 600 µl |
|  | Passive (0 µl) | pAP 100% (0 µl) | 0 µl |
|  |  | pAP 50% (0 µl) | 0 µl |
|  |  | pAP 0% (0 µl) | 0 µl |
| Poor-Safe (150 µl) | Active (200 µl) | Bad-Good (200 µl) | 200 µl |
|  |  | Good (200 µl) | 200 µl |
|  | Passive (100 µl) | pRwd 100% (200 µl) | 200 µl |
|  |  | pRwd 50% (100 µl) | 200 µl or 0 µl |
|  |  | pRwd 0% (0 µl) | 0 µl |
| Poor-Dangerous (100 µl) | Active (200 µl) | Bad-Good (200 µl) | 200 µl |
|  |  | Good (200 µl) | 200 µl |
|  | Passive (0 µl) | pAP 100% (0 µl) | 0 µl |
|  |  | pAP 50% (0 µl) | 0 µl |
|  |  | pAP 0% (0 µl) | 0 µl |

| **Color** |  |  |  |  |  |  |  |  |  |  |  |  |  |
| --- | --- | --- | --- | --- | --- | --- | --- | --- | --- | --- | --- | --- | --- |
| **µl** | **0** | **50** | **100** | **150** | **200** | **250** | **300** | **350** | **400** | **450** | **500** | **550** | **600** |
