# Supplementary material for: Lateral Habenula Responses During Eye Contact in a Reward Conditioning Task
Source: Front Behav Neurosci. 2022 Mar 14;16:815461. doi: 10.3389/fnbeh.2022.815461 (PMC8964066; doi:10.3389/fnbeh.2022.815461)
Supplement: Supplementary file 2 [file Table_2.docx]

**Supplementary Table S2. Theoretical punishment prediction**

| **Face/Scene** | **Action Cue** | **Object** | **Outcome** |
| --- | --- | --- | --- |
| Rich-Safe (0 ms) | Active (0 ms) | Bad-Good (0 ms) | 0 ms |
|  |  | Good (0 ms) | 0 ms |
|  | Passive (0 ms) | pRwd 100% (0 ms) | 0 ms |
|  |  | pRwd 50% (0 ms) | 0 ms |
|  |  | pRwd 0% (0 ms) | 0 ms |
| Rich-Dangerous (25 ms) | Active (0 ms) | Bad-Good (0 ms) | 0 ms |
|  |  | Good (0 ms) | 0 ms |
|  | Passive (50 ms) | pAP 100% (100 ms) | 100 ms |
|  |  | pAP 50% (50 ms) | 100 ms or 0 ms |
|  |  | pAP 0% (0 ms) | 0 ms |
| Poor-Safe (0 ms) | Active (0 ms) | Bad-Good (0 ms) | 0 ms |
|  |  | Good (0 ms) | 0 ms |
|  | Passive (0 ms) | pRwd 100% (0 ms) | 0 ms |
|  |  | pRwd 50% (0 ms) | 0 ms |
|  |  | pRwd 0% (0 ms) | 0 ms |
| Poor-Dangerous (25 ms) | Active (0 ms) | Bad-Good (0 ms) | 0 ms |
|  |  | Good (0 ms) | 0 ms |
|  | Passive (50 ms) | pAP 100% (100 ms) | 100 ms |
|  |  | pAP 50% (50 ms) | 100 ms or 0 ms |
|  |  | pAP 0% (0 ms) | 0 ms |

| **Color** |  |  |  |  |  |  |  |  |  |
| --- | --- | --- | --- | --- | --- | --- | --- | --- | --- |
| **ms** | **0** | **12.5** | **25** | **37.5** | **50** | **62.5** | **75** | **87.5** | **100** |
