# Supplementary figures and images for: Lateral Habenula Responses During Eye Contact in a Reward Conditioning Task
Source: Front Behav Neurosci. 2022 Mar 14;16:815461. doi: 10.3389/fnbeh.2022.815461 (PMC8964066; doi:10.3389/fnbeh.2022.815461)

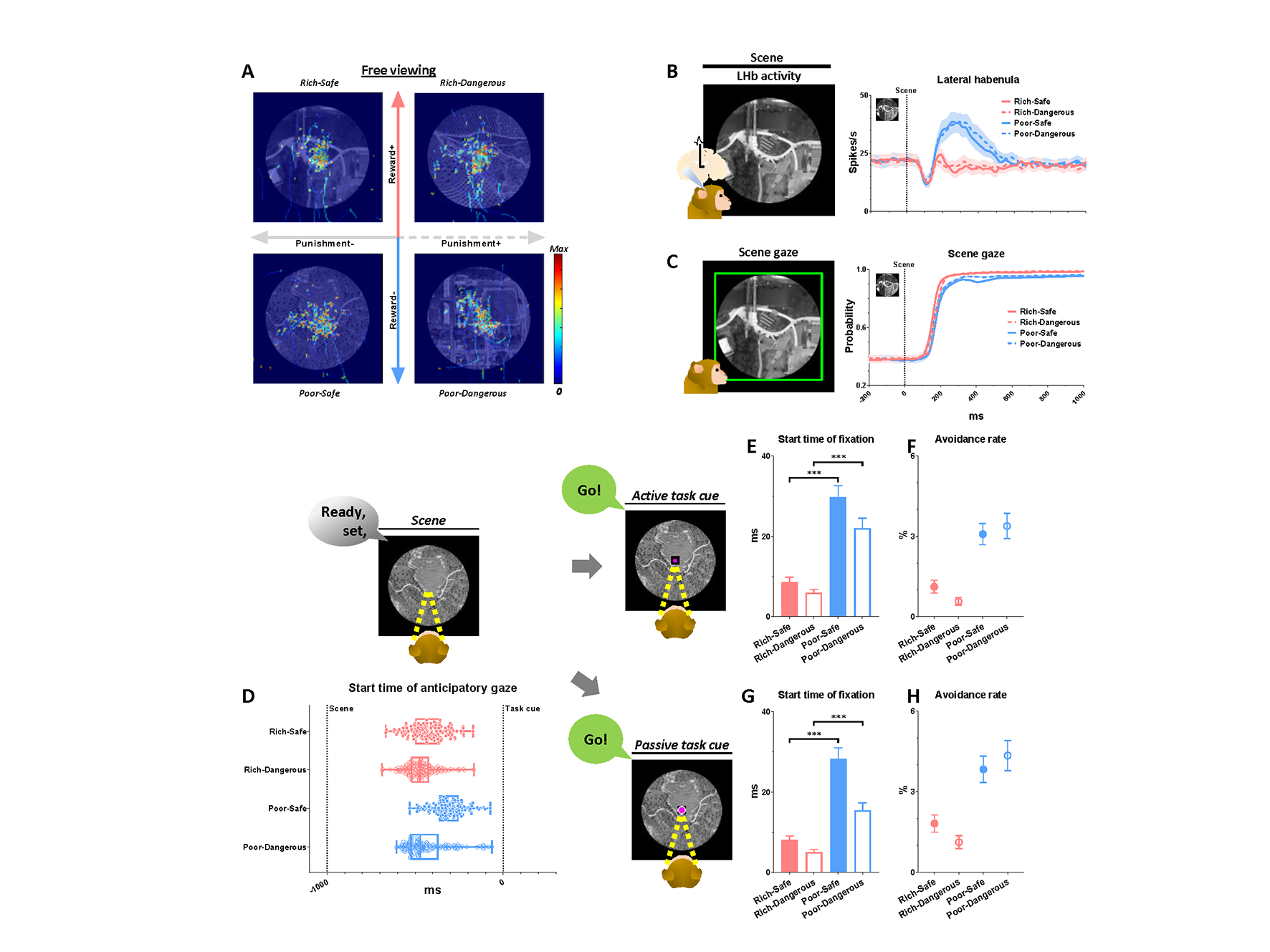

Supplement: Supplementary Figure 1 — Responses to high- and low-valued scenes. (A) Normalized eye scan patterns during the scene-based task procedure. Hot colors indicate a higher gaze probability. (B) Lateral habenula responses to the scenes at the scene onset. (C) Probability of gaze on the scene regions. (D) Start time of anticipatory gaze on the center region before task cue onset. (E) Start time of fixation on the active task cue after the cue onset. (F) The rates of trials that monkeys refused to fixate their gaze on the active cue. (G) Start time of fixation on the passive task cue after the cue onset. (H) The rates of trials that monkeys refused to fixate their gaze on the passive cue. [file Image_1.TIFF]
